# A Surgical Variant of the Pre-Lacrimal Approach to the Maxillary Sinus

**Published:** 2019-09

**Authors:** Andrea Preti, Mario Turri-Zanoni, Alberto-Daniele Arosio, Paolo Castelnuovo, Paolo Battaglia

**Affiliations:** 1 *Department of Medicine and Surgery, Translational and Experimental Medicine Ph. D, University of Insubria, Varese, * *Italy and Department of Otorhinolaryngology, Multimedica, San Giuseppe Hospital, Milano, Italy.*; 2 *Department of Biotechnology and Life Sciences (DBSV), Unit of Otorhinolaryngology, University of Insubria, Azienda Ospedaliero-Universitaria Ospedale di Circolo e Fondazione Macchi, Varese, Italy.*

**Keywords:** Endoscopic endonasal surgery, Maxillary sinus, Osteotomy, Pre-lacrimal approach

## Abstract

**Introduction:**

When dealing with maxillary sinus pathology, some areas of the sinus remain difficult to examine. In this regard, the pre-lacrimal approach is a minimally invasive technique to reach anterolateral areas of the maxillary sinus while preserving the physiological nasal function.

**Materials and Methods:**

The present study aimed to provide technical hints related to pre-lacrimal approach acquired through a large number of performed procedures.

**Results:**

According to the results, the mucosa incision was performed more anteriorly than the osteotomy using the proposed surgical variant. Moreover, this procedure prevented post-operative annoying symptoms related to the possible presence of an inferior meatotomy.

**Conclusion:**

The pre-lacrimal approach to the maxillary sinus should be considered as a part of the surgical armamentarium to address the maxillary sinus.

## Introduction

Some parts of the maxillary sinus are hard-to-reach through standard middle meatal antrostomy. The pre-lacrimal approach was developed to gain access to the most ‘blind’ regions of the sinus, such as the anterolateral and lacrimal recess, as well as anterior and inferior walls.

The disadvantage associated with the utilization of this technique is related to the possible residual inferior meatotomy, which may impair the physiological mucociliary clearance. This complication is attributed to the overlap of the mucosal incision and the osteotomy occurring in the standard procedure. With this background in mind, the purpose of the present study was to propose a surgical variant to the classical pre-lacrimal technique capable of overcoming this problem.

Technical Description

A vertical mucosal incision of the lateral nasal wall was performed more anteriorly than the classical pre-lacrimal approach using a 0.4 mm scope at the level of the pyriform aperture. 

The mucoperiosteum was elevated from the lateral nasal wall along the inferior meatus and gently upward the rotation of the inferior turbinate. Subsequently, a 3-mm osteotomy was performed posteriorly to the pyriform aperture just anteriorly to the lacrimal duct (Fig. 1).

**Fig:**
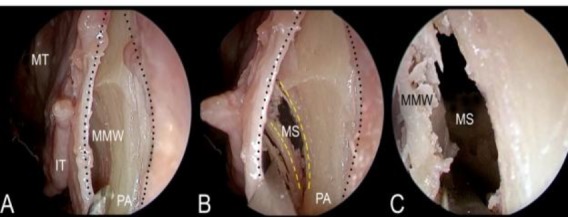
**A**) The mucosal incision is performed at the level of the pyriform aperture (PA). The mucoperiosteum is elevated exposing the medial maxillary wall (MMW). In the picture are also appreciable the inferior (IT) and the middle turbinate (MT). **B**) The osteotomy is performed as in the same position of the classical pre-lacrimal approach. Now it is possible to appreciate the maxillary sinus (MS). **C**) Access to the maxillary sinus

Once the access to the sinus was granted, the visualization of all its walls was possible through a scope with an angle of 45 degrees. The inferior turbinate was relocated downward and the mucosal incision was sutured at the end of the procedure.

## Discussion

The approach to all the walls and recesses of the antrum can be challenging with standard or enlarged middle meatal antrostomy. On the other hand, the complete removal of the medial wall of the sinus (e.g. medial maxillectomy) helps examine all the walls; however, it is accompanied by the physiological dysfunctionality of the inferior turbinate and sectioning the nasolacrimal duct. The pre-lacrimal approach seems to be less invasive and it is similarly effective for the detection of the above mentioned hard-to-reach areas.

Our technical alternative is to perform the mucosal incision more anteriorly than the osteotomy. When the inferior turbinate is sutured back in its original position, it prevents persistent communication between nasal fossa and the sinus in the inferior meatus. Moreover, a strip of the mucosa is left at the level of the pyriform aperture which acts as a ‘hinge’ and promotes the repositioning of the inferior turbinate. 

## Conclusion

The pre-lacrimal approach is a technique that should be considered as part of the surgical armamentarium to address the maxillary sinus. The technical hint described in the present study can help the feasibility of this procedure and prevent the drawbacks of an inferior meatotomy.

